# The Influence of the Mediterranean Dietary Pattern on Osteoporosis and Sarcopenia

**DOI:** 10.3390/nu15143224

**Published:** 2023-07-20

**Authors:** María Carmen Andreo-López, Victoria Contreras-Bolívar, Beatriz García-Fontana, Cristina García-Fontana, Manuel Muñoz-Torres

**Affiliations:** 1 Endocrinology and Nutrition Unit, University Hospital Clínico San Cecilio, 18016 Granada, Spain; mcandreo21@gmail.com (M.C.A.-L.); cgfontana@hotmail.com (C.G.-F.); mmt@mamuto.es (M.M.-T.); 2 Instituto de Investigación Biosanitaria de Granada (Ibs. Granada), 18014 Granada, Spain; bgfontana@fibao.es; 3 CIBER on Frailty and Healthy Aging (CIBERFES), Instituto de Salud Carlos III, 18012 Granada, Spain; 4 Department of Cell Biology, University of Granada, 18016 Granada, Spain; 5 Department of Medicine, University of Granada, 18016 Granada, Spain

**Keywords:** Mediterranean diet, osteoporosis, sarcopenia

## Abstract

Diet is a modifiable factor in bone and muscle health. The Mediterranean diet (MedDiet) is rich in nutrients and contains key bioactive components with probable protective effects on muscle and bone deterioration. Osteoporosis (OP) and sarcopenia are diseases that increase frailty and susceptibility to fracture, morbidity and mortality. Therefore, it is necessary to combat them in the population. In this regard, MedDiet adherence has proven to be beneficial to bone mineral density (BMD), muscle mass, physical function, OP and sarcopenia. Hence, this diet is proposed as a therapeutic tool that could slow the onset of osteoporosis and sarcopenia. However, there is doubt about the interaction between the MedDiet, strength and fracture risk. Perhaps the amount of EVOO (extra virgin olive oil), fruits, vegetables and fish rich in anti-inflammatory and antioxidant nutrients ingested has an influence, though the results remain controversial.

## 1. Introduction

The diet is a modifiable lifestyle factor in relation to bone and muscle health [1,2,3]. The beneficial effects of calcium, vitamin D and protein on bone and muscle have been supported by research [4,5]. However, the benefits of a healthy diet on bone and muscle health go beyond these nutrients [6,7,8]. Healthy diets that are rich in nutrients and bioactive components appear to be key to the ongoing remodeling of the skeleton and slowing down the loss of bone and muscle mass [9]. In fact, the bioactive compounds such as phytochemicals and antioxidants in fruits and vegetables influence bone and muscle metabolism mainly by reducing oxidative stress and inflammation [10]. Therefore, the diet does not cure but may slow down the degeneration of bone and muscle tissue.

**Figure 1:**
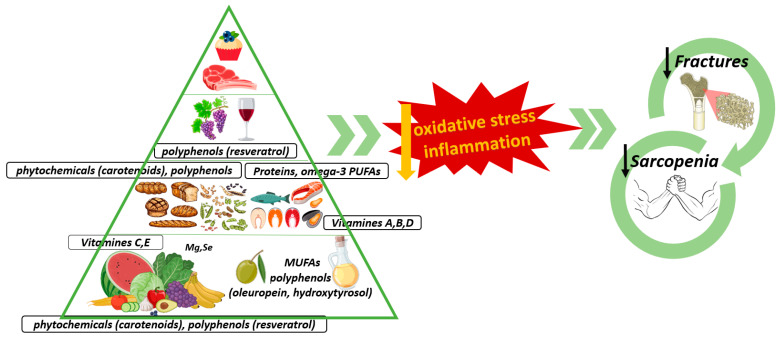
Interplay between Mediterranean diet, osteoporosis and sarcopenia.

Specifically, the Mediterranean diet (MedDiet) is an example of a healthy dietary pattern. The MedDiet is characterized by a high content of fruit, vegetables, whole grains and fish and favors the intake of anti-inflammatory nutrients such as fiber, omega-3, monounsaturated fatty acids (MUFA) and polyphenols of olive oil (OO), which may have a positive influence on the reduction of the bone resorption process and muscle wasting (Figure 1) [6,7].

Currently, osteoporosis (OP) is an important worldwide public health concern. OP is characterized by bone mass loss and trabecular microarchitecture alterations that increase frailty and susceptibility to fracture [11]. Similarly, sarcopenia contributes to the risk of fracture in the population, promoting morbidity and mortality. Namely, sarcopenia is defined as a progressive and generalized skeletal muscle disorder [12]. Moreover, the age-related losses of skeletal muscle and function are risk factors for frailty, OP and fractures [13].

Interestingly, epidemiological studies have demonstrated that the incidence of OP in Europe is lower in the Mediterranean area [14,15]. However, there are no clear data on the incidence of sarcopenia in this area. Nevertheless, adherence to the MedDiet has been associated with improved muscle mass and function [1]. The MedDiet even seems to slow the worsening of frailty due to its important role in the prevention of various chronic diseases.

In addition, it has recently been shown that microbiota may be modified by the diet. Indeed, the human microbiota appears to play a key role in health, as it functions in the interrelationship between diet, medication use, lifestyle, host immune development and health. In fact, a recent study in Spain identified several beneficial bacteria that are more abundant in individuals with greater adherence to the MedDiet, with *Bifidobacterium animalis* being the most abundant [16]. These modifications in the microbiota could play a key role in the regulation of bone metabolism and pathogenesis and prevention [17], as well as in skeletal muscle pathophysiology [18].

Conversely, the relationship between fracture risk and MedDiet is not clear [19]. In addition, there are doubts about the association between the Mediterranean dietary pattern and muscle strength [20,21]. Therefore, the aim of this review was to summarize the evidence of the role the MedDiet plays in OP and sarcopenia prevention and/or improvement in adults and the elderly [7].

## 2. Materials and Methods

A comprehensive search of the literature published in PubMed was conducted to identify articles on the MedDiet, OP and sarcopenia. Search strategies were based on the following search terms: MedDiet, polyphenols, omega-3 poly-unsaturated fatty acids (PUFAs), OO, OP, bone mineral density (BMD), bone, muscle, strength, sarcopenia, falls, hip, spine and fragility fractures. A selection of articles published in English providing original human research, observational prospective and retrospective studies, randomized controlled trials, reviews and meta-analyses were included.

In addition, we considered case series, single-case reports, editorials, research or original articles, letters to the editor, comments (to an article or from the editor), responses (to a comment, letter or article), corrections, short reports, short communications, perspectives, opinions and discussions. Priority was given to the largest studies, the strongest available evidence and the most recent studies.

## 3. The Mediterranean Diet

The MedDiet represents a model of a healthy diet. It contributes to a healthy lifestyle and a better quality of life [22,23]. Interestingly, this diet has traditionally been the basis of food habits around the Mediterranean area for hundreds of years [24].

The MedDiet is closely related to traditional olive growing in the Mediterranean area. In this regard, the MedDiet is characterized by a high consumption of unsaturated fat, whose main source is OO—especially virgin olive oil (VOO) and extra virgin olive oil (EVOO)—and the high intake of vegetables, fruits, legumes, potatoes, bread, and other (unrefined) cereals, nuts and seeds, opting for fresh, seasonal, locally grown and minimally processed foods. Dairy (especially cheese and yogurt) consumption is moderate. Likewise, fish (rich in long-chain PUFAs, particularly omega-3) and poultry are consumed in low or moderate amounts. Also, the consumption of wine at meals is moderate. The Mediterranean dietary pattern involves low consumption of red meat and sweets [24]. Some bioactive compounds in the MedDiet are highlighted for their potential beneficial effects. Between them, vitamins, minerals, polyphenols, fiber, nitrates, PUFAs and MUFAs, in combination or separately [6,7], are included. Among the PUFAs, the essential omega-6 fatty acid is linoleic acid and longer omega-3 PUFAs, such as eicosapentaenoic acid (EPA) and docosahexaenoic acid (DHA), are derivatives of alpha-linoleic acid. These are mainly present in fish oils [25].

The beneficial effects of the MedDiet are numerous. In general, the MedDiet is associated with healthy aging [23]. There is even consistent evidence that adherence to a traditional MedDiet is linked to a reduction in the risk of all causes of mortality, as well as a decrease in mortality from coronary heart disease [26]. Also, this diet prevents and helps to manage chronic degenerative diseases (represented mainly by obesity, cardiovascular diseases, diabetes, chronic kidney diseases, OP, sarcopenia and neurodegenerative diseases) [27].

Therefore, the Mediterranean dietary pattern may be a preventative and therapeutic tool in patients with OP and sarcopenia.

## 4. Relationships between Osteoporosis and Sarcopenia

Bone and skeletal muscle are connected. They are recognized as among the largest tissues in the body and constitute the musculoskeletal system, whose main function is locomotion. At the molecular level, the relationship between bone tissue and skeletal muscle is being increasingly studied. In fact, bone growth and repair have been stimulated by alterations in contractility and myokines secreted by muscle [28]. Moreover, the mechanical and trophic interaction between the bone and muscle compartment is essential for the functionality of living beings [29,30]. Osteopenia/OP and sarcopenia can occur simultaneously (osteosarcopenia) or separately [31].

OP is a systemic skeletal disease characterized by bone mass loss and trabecular microarchitecture alterations that favor fragility and susceptibility to fracture [11]. In fact, symptomatic OP is defined by low-energy fractures of the hip or spine, even with a normal BMD measured by T-score [32]. BMD is usually measured in the lumbar spine and femoral neck by dual energy X-ray absorptiometry (DXA) due to the definition of OP, which includes a T-score below −2.5 SDs (standard deviations referring to peak bone mass in young healthy women) and osteopenia by a T-score between −1 and −2.5 SDs for BMD measured by DXA in the lower spine and/or hip [33]. In addition, recently, the Vertebral Fracture Assessment (VFA), obtained with DXA, has allowed for the detection of vertebral fractures that go undetected by other techniques and that can alter the DXA result, falsely increasing BMD [34]. Compared to conventional X-rays, it produces less radiation and is more comfortable and economical [34]. Another useful tool is the Trabecular Bons Score (TBS), which, unlike DXA, which gives information on the cortical gap and provides information on the state of the trabecular bone. This technique complements DXA in the diagnosis of OP; according to its score, the bone microarchitecture can be classified into a TBS ≥ 1.31, which corresponds to normal microarchitecture, a TBS of 1.23–1.31, which corresponds to partially degraded microarchitecture and a TBS < 1.23, which responds to degraded microarchitecture [35,36,37]. Occasionally, some studies have broadened the BMD analysis with the volumetric parameter bone mineral density (vBMD) (total, trabecular and cortical bone density) for distinguishing trabecular from cortical bone. Other image techniques such as quantitative bone ultrasound (QUS) and peripheral quantitative computed tomography (pQCT) were also used to measure bone status in a few studies [38].

OP is an important worldwide public health problem. It affects 27.5 million people (22 million women and 5.5 million men) aged 50–84 years worldwide, and its prevalence is estimated to be 33.9 million by 2025 [39].

On the other hand, sarcopenia is referred to as a progressive and generalized skeletal muscle disorder. Currently, the most widely accepted definition is that proposed by the European Working Group on Sarcopenia in Older People (EWGSOP). It establishes that a person with low muscle strength and low muscle mass or quality will be diagnosed with sarcopenia. The detection of low physical performance is used to identify severity of sarcopenia [12]. This could be caused by age, chronic disease, physical inactivity, reduced mobility or malnutrition [40].

OP and sarcopenia contribute to the risk of fracture in the population, increasing morbidity and mortality [41,42]. OP is a major risk factor for fragility fractures. Moreover, the age-related loss of skeletal muscle and function is a risk factor for OP and fractures. Hence, a number of other risk factors, such as falls, influence the fracture risk beyond BMD [13]. In fact, low handgrip strength (HGS: a tool used to assess muscle strength) and low femur neck BMD were independent predictors of previous fragility fractures [43]. So, among the modifiable risk factors that can impact on the muscle and bone, diet seems to play an important role. Specifically, a healthy diet such the MedDiet has been associated with a lower number of fragility fractures [2,3,44].

Certainly, diet is not a cure, but it may slow the deterioration of bone and muscle tissue and thereby decrease the risk of falls and fractures [9]. In addition, the microbiome plays a fundamental role as a mediator between muscle, bone and the diet [17,45,46].

## 5. Interplay between Osteoporosis and the Mediterranean Diet

The lower risk of chronic noncommunicable diseases associated with the MedDiet is known. However, the study of the relationship between the MedDiet and bone health, more specifically BMD, is recent [3]. So, current studies point to the potential benefits of the MedDiet for bone health (namely, bone mineral status, bone biomarkers and fracture incidence) [2,3,38]. In fact, several authors indicated that the low incidence of OP in Mediterranean countries could be explained by diet [2]. This diet, high in essential nutrients and other bioactive components (calcium and vitamin D, protein and magnesium (Mg), among others), is involved in maintaining healthy bones. Thus, an optimal and continuous nutrient supply is necessary to compensate for the constant remodeling of the skeleton and muscle wasting due to inflammation and oxidative stress [47]. In this regard, the anti-inflammatory properties of some of its components, such as fruits, vegetables, nuts, cereals, legumes, fish and, in particular, EVOO (as a main source of fat) seem to play a relevant role, decreasing the bone reabsorption process (Figure 1) [7,48,49,50]. Contrarily, the Western or pro-inflammatory dietary pattern, rich in soft drinks, fried foods, meat, processed products, sweets and refined grains, is associated with OP and calcium nephrolitiasis [49,51].

However, the available data correlating the MedDiet with good bone health have been deemed controversial by a few studies. In fact, the association between the MedDiet and a lowered risk of fracture has been called into question [19,52,53].

### 5.1. Bone Mineral Status and Bone Biomarkers

The BMD was the common bone outcome measured in the majority of studies [3,38,54,55]. In fact, several of them related adherence to the MedDiet to higher BMD as well as a reduced risk of fracture [3,55]. These works have been conducted in a postmenopausal and/or elderly population [2,56,57]. However, Pérez-Rey et al. analyzed a population of Spanish premenopausal women and detected a positive association between the degree of adherence to the MedDiet and bone density assessed by DXA, QUS and pQCT [38]. Likewise, in a Chinese middle-aged and elderly population, it was found that greater adherence to the MedDiet was related to higher BMD by DXA [56]. This association was also observed in an American population [2]. Nevertheless, Kotogianni et al. observed no significant effect of the Mediterranean dietary pattern on bone mass. However, a positive relationship was found between MedDiet adherence and bone mass [58]. Similarly, in the NU-AGE (New Dietary Strategies Addressing the Specific Needs of the Elderly Population for Healthy Aging in Europe) multicenter randomized trial, participants aged 66–74 years who ingested a Mediterranean-like dietary pattern and a vitamin D3 supplement (400 IU/d) did not exhibit any effect on the BMD, but individuals with OP had a significantly reduced rate of bone loss of at the femoral neck [54].

The role of certain nutrients present in the MedDiet may have potential benefits for BMD (Table 1). Traditionally, studies have focused on the roles of calcium, vitamin D, protein and dairy, but there is growing evidence of a positive relationship between the components of fruits and vegetables and bone health. In fact, adequate intake of some of these components, such as phytochemicals (e.g., carotenoids, and genistein aglycone), vitamin C and selenium, could improve BMD. Specifically, the study of Marini et al. found that beta-carotene slowed bone reabsorption, and genistein aglycone administration decreased levels of bone reabsorption markers (pyrrolidonyl aminopeptidase (PYR), telopeptide of type I collagen (CTX) and receptor activator of nuclear factor B (RANKL) and increased new bone formation markers (IGF-I and osteoprotegerin (OPG)) [59]. Furthermore, vitamin C has been associated with BMD in the femoral neck and lumbar spine [50,60]. Regarding selenium, higher levels were positively related to site-specific BMD [61]. Similarly, other diets rich in fruits and vegetables, such as DASH (Dietary Approaches to Stop Hypertension), reduced serum osteocalcin by 8–11% and CTX by 16–18% [48].

Along the same lines, the dietary intake of OO has been positively associated with total, trabecular and cortical BMD [62]. Olive polyphenols have been shown to protect bone health via oxidative stress reduction and anti-inflammatory effects (Figure 1). A dose-dependent protective effect of oleuropein (a polyphenol from OO) has been discovered in a model of rats with bone loss [63]. Also, hydroxytyrosol, formed by the hydrolysis of oleuropein during the maturing of olives, could prevent osteopenia by increasing bone formation [64]. In this regard, olive polyphenols favor the growth and differentiation of pre-osteoblasts and decrease osteoclast formation [65,66,67,68]. In addition, Fernandez-Real et al. showed that a MedDiet enriched with VOO, compared with a walnut-enriched MedDiet and a low-fat diet for two years, had a positive effect on serum markers of bone formation and caused a reduction in the circulating concentration of markers of bone reabsorption [69].

Another phenolic compound that belongs to the group of phytoestrogens and is present in vegetables and abundant in red grapes and red wine is resveratrol (RES). This compound promotes the maturation of osteoblasts and slows down the formation of osteoclasts in vitro. Hence, RES has been related to the preservation of bone mass, microarchitecture and strength in rats [70]. In fact, moderate alcohol intake, specifically red wine, a typical component of the MedDiet, might prevent bone loss in older men, and a moderate intake of wine might do so in women [71,72].

In addition, moderate fish intake is a feature of the MedDiet. Fish contains numerous nutrients that have a beneficial effect on bones. Specifically, it is a source of high-quality protein, omega-3 fatty acids, vitamins A and D, and minerals such as selenium, calcium, iodine and zinc [53]. In fact, the recent review by Perna et al. indicates that fish has a positive impact on BMD mainly due to the anti-inflammatory effect of omega-3 fatty acids. In particular, DHA are positively related with bone mineral accumulation and with peak BMD in young men. DHA seems to be a vital constituent of healthy modeling bone because it accumulates in the osteoblast-rich periosteum of the femur in growing rats [10,73]. Moreover, omega-3 fatty acids favor duodenal calcium absorption and lead to decreased calcium excretion [10,74].

Also, the intake of legumes and cereals (grains) is high in the Mediterranean dietary pattern. These foods are rich in vitamin B, calcium and phytochemicals. Their adequate consumption has been related to higher BDM and decreased bone loss with aging. Therefore, the data suggest that legumes and grains may protect bone metabolism in humans [75,76].

In contrast to the Mediterranean dietary pattern, a higher adherence to pro-inflammatory dietary patterns, distinguished by its content in processed food, fats and red meat, has been linked to lower BMD and increased peripheral inflammation [49].

### 5.2. Osteoporotic Fractures

Different strategies to decrease the increase in fragility fractures have been sought. Among them, adherence to a healthy diet such as the MedDiet was linked to a lower rate of hip fractures [2,44]. This reduction in the number of hip fractures was also observed in Mediterranean population or Americans who follow the Mediterranean dietary pattern compared to the rate of fractures in the population of Northern Europe [2,77]. According to Haring et al., a lower risk of hip fractures was reported with higher adherence to the MedDiet in women aged 50–79 years in a post hoc analysis of longitudinal data from the United States Women’s Health Initiative (median follow-up of 15.9 years) [2]. Similarly, the EPIC (European Prospective Investigation into Cancer and Nutrition) study in men and women (*N* = 188,795 participants, 802 incident hip fractures) observed that a higher adherence to the MedDiet reduced the incidence of hip fractures by 7%, particularly in men [52]. Also, the meta-analysis of Malmir specified that higher adherence to the MedDiet was associated with a 21% decreased hip fracture risk [3].

To explain the inverse association between MedDiet adherence and hip fractures, whether or not type 2 diabetes mellitus (T2DM) and body mass index (BMI) mediated this relationship was analyzed. The state of systemic inflammation that encourages the development of chronic noncommunicable diseases such as OP, obesity and T2DM [78,79], could play a role. In fact, Cauley et al. observed that baseline markers of inflammation were higher among subjects who subsequently experienced an incident fracture [80]. However, Mitchell et al. reported a direct effect on hip fracture risk via pathways other than T2DM and BMI, yet they could not rule out the mediating effects of BMI and T2DM, even if their effects canceled each other out [81].

On the other hand, better MedDiet adherence in middle-aged women (45–65 years), with or without OP, was associated with a lower bone risk fracture [82]. However, the evidence of a protective effect of the MedDiet on total fracture risk has been contradictory. In fact, a small older French population study found that higher MedDiet adherence was associated with a nonsignificant reduced risk of fractures to the hip, spine and wrist [19].

Nevertheless, it is necessary to emphasize that causality has not been established by these studies, as they are not randomized trials; most are cross-sectional and observational. In addition, the effect of the MedDiet is possibly due to particular dietary components whose intake should be increased. For example, in the PREDIMED (Prevention with Mediterranean diet) trial, greater consumption of EVOO was associated with a lower risk of OP-related fractures in an older Mediterranean population at high cardiovascular risk [7]. Specifically, individuals in the highest tertile of EVOO consumption had a 51% lower risk of fractures than those in the lowest tertile. Total and standard OO intake has not been associated with fracture risk [7]. Therefore, EVOO is the best quality oil compared to other OOs. Indeed, EVOO has the highest amounts of bioactive and antioxidant components, such as polyphenols, with probable beneficial effects on bone metabolism (Figure 1) [83]. In contrast, regular OO is more than 80% refined, with fewer antioxidant and anti-inflammatory compounds [7]. Also, fish, a source of omega-3 PUFA, has been inversely associated with hip fracture risk [84]. Other typical Mediterranean foods have been demonstrated to reduce the risk of fracture, such as legumes, cereals [76] and wine [82]. Also, high fruit and vegetable intake can give antioxidant power due to vitamins such as vitamin C. Specifically, this is required for collagen synthesis and osteoblast generation. In this regard, a higher vitamin C intake has been related to higher BMD at the femoral neck and lumbar spine and a lower fracture risk [9,85]. In addition, a dose–response relationship between fruit and vegetable intake and hip fracture has been described. Hence, consumption of fewer than five servings of fruit and vegetables per day was associated with higher rates of hip fracture [86]. Also, the combination of fruit and vegetables with fermented milk (yogurt or sour milk) was shown to decrease the rate of hip fracture [87].

In contrast, supplementing the diet with sugar does not promote bone health. In fact, a substudy of PREDIMED, PREDIMED-Reus detected that a higher dietary glycemic index and higher dietary glycemic load values could increase the risk of osteoporotic fracture [88].

On the other hand, Jennings et al. reported that the additive effects of individual components within the Mediterranean diet in a non-Mediterranean region have a greater effect on fracture incidence than the individual components of this diet [54].

In summary, different factors have to be considered. However, adherence to a Mediterranean dietary pattern with an adequate consumption of OO, preferably EVOO, fish, fruit and vegetables, avoiding high-glycemic-index sugars, could decrease the risk of fractures related to OP (Table 1).

## 6. Interplay between Sarcopenia and the Mediterranean Diet

The loss of skeletal muscle mass and strength or sarcopenia contributes to the risk of frailty, fractures and death. The influence of endocrine interactions between skeletal muscle and bone, muscle force-generated mechanical signals and age-related molecular mechanisms probably contributes to the deterioration of muscle function and mass, such as via low-grade inflammation and oxidative stress. These act as a trigger for protein catabolism and lead to protein turnover in skeletal muscle [89,90]. Therefore, chronic inflammation aggravates muscle loss [91]. In fact, increased inflammatory cytokines have been associated with lower HGS, indices of skeletal muscle mass or physical performance and disability [8]. To slow bone loss and muscle wasting, thus preventing fragility fractures, nutrient-dense and bioactive components of an healthy diet are essential [9]. In this regard, adherence to the MedDiet has been positively associated with muscle mass and function [1], while the results related to muscle strength were less clear [20,21]. It has even been shown to promote the preservation of skeletal muscle due to micronutrients (vitamins C and E, omega-3 PUFA, Mg, polyphenols and carotenoids) with their potential anti-inflammatory and anti-oxidant properties or to play a direct role in muscle metabolism such as with Mg (Figure 1) [8,90]. Furthermore, higher MUFA intake and adherence to the MedDiet has been related to fewer falls in community-dwelling older men [6].

### 6.1. Strength, Muscle Mass and Function

According to the EWGOSP, low muscle strength measured by HGS [12] is useful to denote the risk of sarcopenia. In addition, low muscle mass confirms sarcopenia [12]. Finally, muscular function is evaluated with several tests such as sitting time, Short Physical Performance Battery (SPPB), etc. These data are used to define the severity of sarcopenia [12].

Specifically, Barrea et al. have studied the influence of the Mediterranean dietary pattern on HGS and found positive results in an active elderly female population [1]. Also, Mendes et al. showed a positive association between the MedDiet and HGS in an older population. Specifically, fruit, nuts, EVOO and wine rich in several antioxidant micronutrients were associated with HGS (Table 1) [1,92]. In contrast, some works have not linked the MedDiet to HGS [20,21]. These differences between studies could probably be due to the amount of foods rich in carotenoids, vitamins and minerals consumed by the study sample. For example, Mendes et al. found that individuals who consumed ≥3 servings of fruits per day, according to the PREDIMED, had a 50% reduced risk of presenting with low HGS compared to those who did not consume <3 servings of fruits per day [92]. In fact, lower plasma levels of carotenoids, a marker of poor fruit and vegetable intake, have been linked to low skeletal muscle strength [93]. Fruits also contain vitamins C and E, which have beneficial effects on muscle metabolism [94,95]. In addition, individuals with higher levels of fiber and PUFA in their diet presented higher HGS after adjusting for confounding factors [96]. Curiously, the food groups that were associated with HGS were also related to sitting time [92]. Thus, the interrelationship between HGS and sitting time is reflected. It is known that physical activity favors muscle mass and strength, and the latter promotes functionality [97].

A few studies have been carried out on the influence of the Mediterranean diet on muscle mass, but none of these was a clinical trial (Table 1) [6,98,99]. In the study of Cervo et al., higher MedDiet adherence was associated with higher appendicular lean mass [6]. The study of Isanejad et al. observed that higher MedDiet adherence was related to lower skeletal muscle index and lean mass loss [98]. Finally, in Kelaiditi et al., FFM% (fat-free mass/weight × 100) by DXA was used to measure muscle mass and the influence of the MedDiet. They detected a positive association between the MedDiet and muscle mass in healthy women. Moreover, it was found that grains, fruit and vegetables were positively and significantly related [99]. Interestingly, these foods are rich in Mg, which plays a relevant role in musculoskeletal health and is important in establishing prevention strategies for sarcopenia, OP and fractures [8]. In fact, the association with Mg and FFM% was three times greater than protein [8]. Nevertheless, protein intake is necessary for muscle health [100]. However, the role of protein origin, animal or plant, is not clear. In fact, a recent meta-analysis found that animal and plant proteins were equivalent in their effects on lean mass or muscle strength [91].

Namely, whole grains contain bioactive compounds such as polyphenols and carotenoids that contribute to muscle anabolism [101]. In addition, fruits and vegetables decrease oxidative stress and the inflammatory burden, which are implicated in the etiopathology of sarcopenia due to carotenoids, antioxidant vitamins and phytochemicals (Figure 1) [51,102,103]. In fact, oxidative stress decreases muscle mass because it favors proteolysis and reduces muscle anabolism [102]. Moreover, fruit and vegetables alkalinize the acid–base load of the diet. Indeed, meat is a major contributor to this load, and its intake is negatively associated with FFM% [99].

With respect to fats, Welch et al. identified a relationship between the FFM index and the P/S (total PUFA to saturated fat) ratio [90]. In this regard, fish is a source of omega-3 PUFAs, lean proteins, selenium and vitamin D, which all have anti-inflammatory potential. In particular, omega-3 PUFAs activate the mTOR/p70s6k (mammalian target of rapamycin/ribosomal protein kinase S6) signaling pathway [104]. This is a relevant pathway influencing skeletal muscle mass, particularly under mechanical stimulation. Also, EVOO, the main source of fat in the MedDiet, specifically contains polyphenols that have been related to anabolic muscle response and skeletal muscle protein synthesis [105].

On the other hand, the Mediterranean dietary pattern has been shown to positively influence muscle function in several studies [20,92,98]. In this sense, the benefits of the bioactive components of fruit were reflected in the studies. According to Mendes et al., individuals who consume ≥3 pieces of fruit per day, according to the PREDIMED, have a 60% reduced risk of presenting with a longer sitting time, compared to those who did not consume ≥3 servings of fruit per day [92]. Moreover, lower serum levels of vitamin E have been related to reduced physical performance [20]. With respect to PUFA (another star component of the Mediterranean dietary pattern), a lower than average intake of EPA and DHA was significantly associated with poor functional mobility in men aged ≥85 years in the Tokyo Oldest Old Survey on Total Health [106].

### 6.2. Frailty and Falls

Frailty is a state of vulnerability secondary to multisystemic impairment that predisposes to poor resolution of homeostasis following a stressful event [107]. Furthermore, the coexistence of sarcopenia and OP is typical of frail elderly population [33].

An adequate intake of energy, protein, micro- and macronutrients is considered crucial to prevent the onset and progression of frailty [108,109]. In fact, the MedDiet seems to indirectly slow the development of frailty due to its important role in the prevention of various chronic diseases. Specifically, higher MedDiet adherence was associated with a lower likelihood of prefrailty and frailty in a sample of 13,100 postmenopausal Finnish women aged 65 years or older [110]. Greater acceptance of the MedDiet was associated with a 62% lower risk of frailty in an elderly French sample [111]. Moreover, higher consumption of vegetables (such as legumes and nuts, mushrooms and vegetable products) and fruit was associated with a lower probability of frailty [110]. Moreover, the antioxidant and anti-inflammatory power (due to vitamin C, vitamin E, carotenoids and selenium) of these foods could combat oxidative stress (Figure 1) [112,113] because this mechanism is recognized as an important underlying factor of frailty among the elderly [114]. Moreover, Fougere et al. showed that low serum concentrations of vitamin E are related to frailty, reduced physical performance and disability [20].

Other nutrients of the MedDiet, unsaturated fatty acids, may also contribute to anti-inflammatory properties. For example, higher MUFA intake and adherence to a MedDiet have been related to fewer falls in community-dwelling older men [6]. In contrast, ultraprocessed foods, alcohol and red meat have been linked to sarcopenia and frailty [98,115,116].

## 7. The Microbiome: A Moderator between Diet, Bone and Muscle Health

Approximately 60% of the variation in gut microbiota is linked to diet [117]. Specifically, adherence to the Mediterranean dietary pattern may modulate the microbiota, promoting bone and muscle health [17,45,46]. Preliminary studies linked the MedDiet to an increased number of beneficial microbiota species [118]. Mostly, these bacteria produce short-chain fatty acids (SCFA), such as butyrate, as a result of the fermentation of indigestible carbohydrates. Furthermore, these SCFAs have crucial physiological activities, modulating the metabolic response at various organ sites such as bone and muscle [17,18,45].

Among SCFAs, butyrate is key to the activation of regulatory pathways, which are critical for the anabolic action of PTH in bone [119]. Moreover, a butyrate-producing microbiota appears to influence the bioavailability of vitamin D, a key factor in bone metabolism. Specifically, higher levels of the vitamin D-active hormone calcitriol were detected among participants more likely to possess butyrate-producing bacteria [120]. The MedDiet, rich in fiber, legumes, vegetables, fruit and nuts, was associated with an increase in butyrate-producing taxa such as *Oscillospira* (*Flavonifractorplautii*) [16]. In fact, a recent Spanish study identified several beneficial bacteria that are more abundant in individuals with greater adherence to the MedDiet, with *Bifidobacterium animalis* being the most abundant [16]. Moreover, Takimoto et al. suggested that increased *Bifidobacterium* could favor BMD by decreasing the production of cytokines that regulate bone resorption [121]. Some bioactive components of the MedDiet, such as polyphenols and specifically isoflavones, even reduced bone loss in the lumbar spine, femoral neck and trochanter of 85 postmenopausal women with osteopenia [122].

On the other hand, the ability of the gut microbiome to modulate function and muscle mass has been established [18]. The intestinal microbiota influences skeletal muscle cells by producing or modifying basic substrates for muscle protein anabolism [123]. Specifically, bacterial metabolites such as SCFA have a beneficial impact on muscle physiology [18]. In fact, SCFA appear to decrease inflammation and play a key role in the preservation of skeletal muscle mass [124,125]. Hence, Nikkhah et al. concluded that SCFA-producing species have protective effects against muscle atrophy [45]. Mainly, a reduction in SCFA-producing bacteria (such as from the Lachnospiraceae family) has been detected in age-related sarcopenia. In contrast, the Enterobacteriaceae family, with proinflammatory potential, was increased in animals with muscle atrophy [126]. Interestingly, *Faecalibacterium prausnitzii* of the Oscillospiraceae family, as the main SCFA-producing bacterial group, has been reported to decrease in frail patients [126]. Also, the NU-AGE project associated adherence to the MedDiet for one year with specific alterations in the microbiome. These changes were associated with several markers of reduced frailty and improved cognitive function, and were negatively associated with inflammatory markers [127].

## 8. Conclusions

In general, MedDiet adherence plays a positive role in terms of promoting BMD, muscle mass and physical function, and preventing OP and sarcopenia. This diet is, therefore, proposed as a therapeutic tool that could slow down the onset of osteoporosis and sarcopenia. However, the relationship between this diet and strength or risk of fracture is questioned. The effect of the MedDiet on muscle and bone may possibly be influenced by the quantity and quality of some constituent foods such as EVOO, fruit, vegetables and fish due to their anti-inflammatory and antioxidant properties. These foods could even mediate the microbiome and modulate the beneficial effect on bone and muscle health. Specifically, adherence to the MedDiet could promote the formation of bacterial metabolites such as SCFAs, with positive consequences for bone and muscle physiology. Understanding the mechanisms underlying the gut–bone and gut–muscle axes may be useful in addressing BMD loss and muscle wasting.

Nevertheless, there are limitations to this review. Mainly, most of the data presented are based on cross-sectional and prospective studies. Therefore, it is not possible to establish causality. In fact, clinical trials are needed to reach cause–effect conclusions as to the importance of the adoption of the Mediterranean diet in the prevention and treatment of osteoporosis and sarcopenia in Mediterranean and non-Mediterranean populations.

## Figures and Tables

**Table 1:** Influence of key MedDiet foods on bone and muscle health.

Typical Foods of the Meddiet	BMD	Osteoporotic Fractures	Strength	Muscle Mass
EVOO	+	+	+	+
Vegetables and Fruits	+	+	+	+
Legumes	+	+	Unknown	Unknown
Grains	+	+	Unknown	+
Nuts	Unknown	Unknown	+	Unknown
Fish	+	+	+	+
Wine	+	+	+	Unknown

BMD (Density Mineral Bone) and EVOO (extra virgin olive oil).

## Data Availability

Not applicable.
